# Dietary Magnesium Alleviates Experimental Murine Colitis through Modulation of Gut Microbiota

**DOI:** 10.3390/nu13124188

**Published:** 2021-11-23

**Authors:** Federica Del Chierico, Valentina Trapani, Valentina Petito, Sofia Reddel, Giuseppe Pietropaolo, Cristina Graziani, Letizia Masi, Antonio Gasbarrini, Lorenza Putignani, Franco Scaldaferri, Federica I. Wolf

**Affiliations:** 1 Multimodal Laboratory Medicine Research Area, Unit of Human Microbiome, Bambino Gesù Children’s Hospital, IRCCS, 00147 Rome, Italy; federica.delchierico@opbg.net (F.D.C.); sofia.reddel@opbg.net (S.R.); 2 Dipartimento di Medicina e Chirurgia Traslazionale, Fondazione Policlinico Universitario A. Gemelli IRCCS—Università Cattolica del Sacro Cuore, 00168 Rome, Italy; valentina.trapani@unicatt.it (V.T.); valentina.petito@unicatt.it (V.P.); giuseppe.pietropaolo@uniroma1.it (G.P.); graziani.cristina@gmail.com (C.G.); letizia.masi94@gmail.com (L.M.); antonio.gasbarrini@policlinicogemelli.it (A.G.); 3 CEMAD—IBD UNIT—Unità Operativa Complessa di Medicina Interna e Gastroenterologia, Dipartimento di Scienze Mediche e Chirurgiche, Fondazione Policlinico Universitario “A. Gemelli”, IRCCS, 00168 Rome, Italy; 4 Unit of Microbiology and Diagnostic Immunology, Unit of Microbiomics and Multimodal Laboratory Medicine Research Area, Unit of Human Microbiome, Department of Diagnostic and Laboratory Medicine, Bambino Gesù Children’s Hospital, IRCCS, 00165 Rome, Italy; lorenza.putignani@opbg.net; 5 Saint Camillus International University of Health Sciences-UniCamillus, 00131 Rome, Italy

**Keywords:** *Bifidobacterium*, dextran sodium sulfate, inflammatory bowel disease, dysbiosis, Enterobacteriacee, magnesemia, magnesium supplementation, metabolism

## Abstract

Nutritional deficiencies are common in inflammatory bowel diseases (IBD). In patients, magnesium (Mg) deficiency is associated with disease severity, while in murine models, dietary Mg supplementation contributes to restoring mucosal function. Since Mg availability modulates key bacterial functions, including growth and virulence, we investigated whether the beneficial effects of Mg supplementation during colitis might be mediated by gut microbiota. The effects of dietary Mg modulation were assessed in a murine model of dextran sodium sulfate (DSS)-induced colitis by monitoring magnesemia, weight, and fecal consistency. Gut microbiota were analyzed by 16S-rRNA based profiling on fecal samples. Mg supplementation improved microbiota richness in colitic mice, increased abundance of *Bifidobacterium* and reduced Enterobacteriaceae. KEEG pathway analysis predicted an increase in biosynthetic metabolism, DNA repair and translation pathways during Mg supplementation and in the presence of colitis, while low Mg conditions favored catabolic processes. Thus, dietary Mg supplementation increases bacteria involved in intestinal health and metabolic homeostasis, and reduces bacteria involved in inflammation and associated with human diseases, such as IBD. These findings suggest that Mg supplementation may be a safe and cost-effective strategy to ameliorate disease symptoms and restore a beneficial intestinal flora in IBD patients.

## 1. Introduction

Inflammatory bowel disease (IBD) is a chronic relapsing-remitting disease that requires lifelong treatment. The pathogenesis of IBD is still unclear, but it is thought to result from a complex interplay between genetic variability, the host immune system and environmental factors, most prominently diet [1]. In addition to affecting host immunity and intestinal barrier function, dietary nutrients shape our gut microbiota by modulating the abundance of specific species and their individual or collective functions, ultimately affecting host (patho)physiology [2]. IBD is clearly associated with dysbiosis, and gut microbiota modulation is a current objective of IBD treatment, via either supplementation with pro/prebiotics, or fecal transplantation [3,4].

Conversely, nutritional deficiencies are frequent in IBD patients and can have long-term effects on disease course and quality of life [5]. We recently showed that IBD patients present with a substantial magnesium (Mg) deficit, which associates with disease severity, and that dietary Mg supplementation may reduce inflammation and restore normal mucosal function [6]. Despite the abundance of data supporting the immunomodulatory properties of Mg [7], the underlying mechanisms remain poorly described, and the possibility that Mg may affect the gut microbiota is emerging.

Mg homeostasis is of paramount importance in bacteria, and many bacterial species harbor sensor proteins that respond to changes in extracellular Mg by modifying the activity of cognate DNA-binding regulatory proteins [8]. These DNA-binding proteins, in turn, elicit a transcriptional response that helps the microorganism cope with altered Mg availability. The first identified example of a biological system that responds to Mg as its primary signal was the PhoP/PhoQ system, which is present in several enteric bacteria (e.g., *Escherichia coli*, *Salmonella enterica*, *Shigella flexneri*) and some gram-negative species outside of the Enterobacteriaceae family [9]. Low Mg levels activate the PhoP/PhoQ system, which results in the positive regulation of many metabolic processes, including Mg transport, peptidoglycan remodeling, lipopolysaccharide (LPS) modification and various stress-protective functions. Collectively, the pathways activated by low Mg availability lead to increased growth, virulence and survival within macrophages [10].

Sparse reports, mainly from rodent models, have suggested that Mg deficiency can lead to dysbiosis and pathogenic phenotypes [11,12,13,14]. Vice versa, Mg supplementation was shown to positively influence gut microbiota composition and function in mice [15,16]; however, supplementation was provided in the form of mineral-rich extracts, in which Mg was not the only micronutrient.

In view of these premises, we decided to investigate whether an altered composition of gut microbiota, and possibly a shift in microbial function, might be underlying the effect of dietary Mg in our murine model of IBD.

## 2. Materials and Methods

### 2.1. Animals

All procedures involving animals and their care conformed to the Directive 2010/63/EU of the European Parliament. The studies were approved by the Italian Ministry of Health, and by the internal Ethics Committee for Animal Research Studies at Università Cattolica del Sacro Cuore, Rome, Italy (protocol number SF46350/13(NN42), approved on 25 November 2013). Female 7- or 8-week-old C57BL/6 mice (Charles River Laboratories Italia) were housed in a controlled environment (23 °C, 12 h/12 h light/dark, 50% humidity, ad libitum access to food and water). After a 2-week acclimation period, mice were randomized into 3 groups and fed a semi-purified, mineral-adjusted diet containing different amounts of Mg: Hypo-Mg diet (30 mg/kg Mg; *n* = 9), CTRL diet (1000 mg/kg Mg; *n* = 8), and Hyper-Mg diet (4000 mg/kg Mg; *n* = 10), as verified by atomic absorption spectroscopy [6]. Experimental colitis was induced in each diet group by administration (ad libitum in water bottles) of 2.5% *w*/*v* DSS (36-44 kDa molecular weight, MP Biomedicals) dissolved in de-ionized water from day 0 until day 5. From day 5 to 12, DSS was removed, and mice were allowed to recover. For each animal, weight and fecal consistency were evaluated daily, and a disease activity index (DAI) was derived [6]. On the day of sacrifice, animals were anesthetized by intraperitoneal injection, and blood was collected by puncturing the heart. Mice were then sacrificed through cervical dislocation. Magnesemia was measured by atomic absorption spectroscopy, as previously detailed [6].

### 2.2. Bacterial DNA Purification, Amplification, and Sequencing

Stool samples were collected from each mouse on the day of sacrifice and immediately frozen at −80 °C, until analysis. DNA was extracted from 200 mg of stool sample by QIAmp Fast DNA stool mini kit (Qiagen, Germany). Bacterial amplicon libraries (630 bp) were obtained by the amplification of the V3-V4 region of the 16S rRNA gene, using primers reported in the MiSeq rRNA Amplicon Sequencing protocol (Illumina, San Diego, CA, USA) [17]. Negative and positive controls were used to monitor and exclude eventual external and internal contaminations. The sequencing was performed on an Illumina MiSeqTM platform (Illumina, San Diego, CA, USA), where paired-end reads of 300 base-length were generated.

Raw sequences were filtered for their quality and read length by Trimmomatic v. 0.36 software [18]. Then, sequences were filtered for chimera presence by ChimeraSlayer using QIIME 1.9.1 software [19]. Reads were clustered into Operational Taxonomic Units (OTUs) at 97% identity by UCLUST [20] against the Greengenes 13.8 database [21]. MicrobiomeAnalyst [22,23] was used to calculate α- and β-diversity and statistical tests (Mann-Whitney U, Kruskal-Wallis, Benjamini-Hochberg tests, co-occurrence network) on OTUs relative abundances. Data were scaled with total sum scaling method, without any previous rarefaction procedures. Pearson’s correlation between clinical parameters and OTUs was computed by SPSS v.20 software (IBM statistics, Armonk, NY, USA).

To identify OTUs with significantly different abundances between categories, Linear Discriminant analysis (LDA) effect size (LEfSe) analyses was used [24]. An alpha value of 0.05 and an effect size threshold of 2 were used to identify the significant predicted microbial biomarkers.

PICRUSt v1.1.0 tool was applied to predict metagenome functional content from 16S rRNA gene surveys [25]. The resulting function prediction was analyzed by HUMAnN2 v0.99 program to obtain the KEGG (Kyoto Encyclopedia of Genes and Genomes) pathways (http://huttenhower.sph.harvard.edu/humann2) (accessed on 1 November 2021). [26].

All raw sequences have been archived in NCBI database: PRJNA753047 (https://www.ncbi.nlm.nih.gov/bioproject accessed on 1 November 2021)

## 3. Results

### 3.1. Dietary Magnesium Modulates Experimental Colitis

As previously reported [6], for each animal, weight loss and fecal consistency were evaluated daily and converted to a disease activity index (DAI) ranging from 0 (no disease, i.e., no weight loss or normal fecal consistency) to 4 (severe disease, i.e., weight loss higher than 20% or watery diarrhoea). DAI values for weight loss and fecal consistency at sacrifice (day 12) are reported in Figure S1. As expected, in the absence of colitis (no DSS category), no significant differences were found in either weight loss (Figure S1A) or fecal consistency (Figure S1B) among the three diet groups. In DSS-treated (colitic) mice, the Hypo-Mg group displayed the most severe disease, as evidenced by the highest DAI value for both weight loss and fecal consistency, with significant differences in comparison with any of the other groups. Interestingly, Hyper-Mg mice had lower DAI values than CTRL mice, although the difference did not reach statistical significance. These results are consistent with the hypothesis that an Mg-deficient diet exacerbates colitis, whereas Mg supplementation may exert a protective effect.

### 3.2. Dietary Magnesium Modulates Intestinal Microbiota

#### 3.2.1. Microbiota Composition

**Figure 1:**
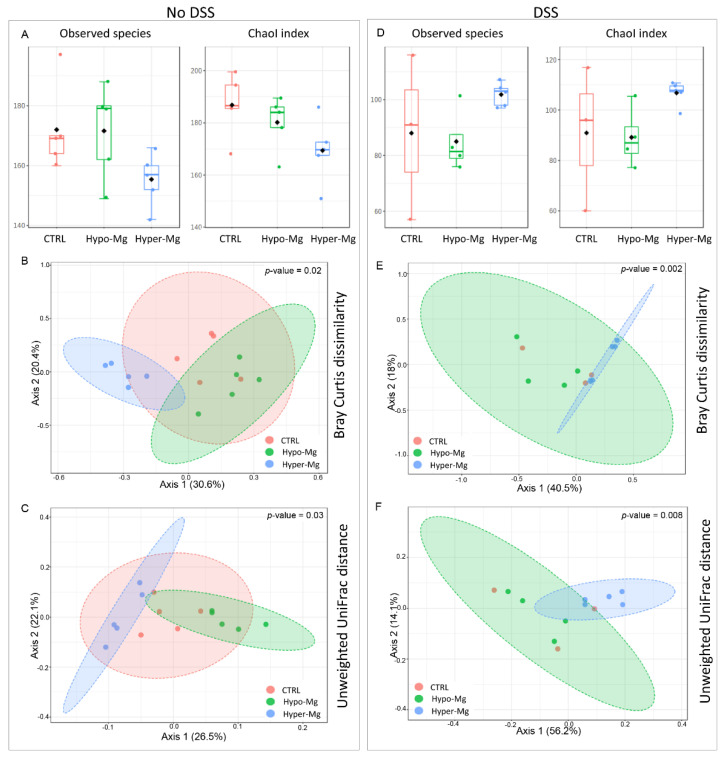
Dietary Mg enriches and shapes microbiota composition in colitic mice. Microbiota α-diversity was evaluated by the Observed species and Chao1 indexes in untreated (**A**) and DSS-treated (**D**) mice on the three Mg-adjusted diets on day 12 (Kruskal-Wallis test *p*-value > 0.05). Principal coordinate analysis (PCoA) plot of bacterial β-diversity was performed on the basis of Bray Curtis dissimilarity (**B**,**E**) and unweighted UniFrac phylogenetic distance (**C**,**F**). The plots show the first two principal coordinates (axes) of PCoA for the three diet groups in untreated (left panel) or DSS-treated (colitic, right panel) mice. PERMANOVA *p*-values are reported in panels (**B**,**C**,**E**,**F**).

A total of 1,067,644 sequencing reads were obtained from 27 samples, with a mean value of 39,542 sequences per sample. Out of 2049 total identified OTUs, 1350 OTUs had ≥2 counts, of which 1199 were filtered out for their low prevalence and low variance, releasing 151 OTUs, further used for relative abundance comparisons. To describe the existing microbial biodiversity within the same group, we measured α-diversity indexes in the three diet groups in untreated (no DSS) (Figure 1A) or DSS-treated (colitic) mice (Figure 1D). Specifically, we analyzed the observed species index, which counts the number of unique OTUs in a group, and the Chao1 index, which is based upon the number of rare OTUs found in a sample.

Although no statistical differences between pairs of diet groups were found, CTRL and Hypo-Mg groups showed a higher number of observed species and higher presence of rare bacterial species than the Hyper-Mg group (Kruskal-Wallis *p*-value > 0.05) (Figure 1A). In DSS-treated (colitic) mice, the trend was inverted: Hyper-Mg and CTRL groups showed higher values of α-diversity indexes than Hypo-Mg mice (Kruskal-Wallis *p*-value > 0.05) (Figure 1D).

β-diversity was analyzed to highlight the differences among different diet groups (Figure 1B–F). Bray Curtis dissimilarity quantifies compositional dissimilarity on the basis of OTU abundance, while unweighted UniFrac phylogenetic distance takes into account the phylogenetic relatedness of OTUs.

β-diversity analysis revealed that, in the absence of colitis, the three diet groups resulted clearly separated (PERMANOVA on Bray Curtis dissimilarity *p*-value = 0.018; on Unweighted UniFrac phylogenetic distance *p*-value = 0.03; Figure 1B,C, respectively). In the presence of colitis, the separation between Hyper-Mg and Hypo-Mg groups was evident, while CTRL mice resulted intermixed with the other groups (PERMANOVA on Bray Curtis dissimilarity *p*-value = 0.002; on Unweighted UniFrac phylogenetic distance *p*-value = 0.008, Figure 1E,F respectively).

**Figure 2:**
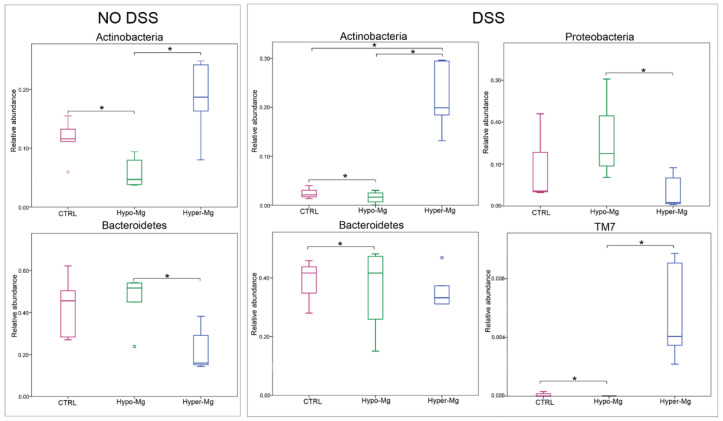
Dietary Mg content modulates gut abundance of specific bacterial phyla. Box plots of the relative abundance of phyla differentially distributed in untreated (**left panels**) and DSS-treated (colitic, **middle** and **right panels**) mice on the three Mg-adjusted diets. Asterisks indicate statistically differential comparisons (Mann-Whitney U *p*-values < 0.05).

At the phylum level, gut microbiota of Mg-deficient mice showed lower relative abundance of Actinobacteria (see Hypo-Mg vs. CTRL and Hyper-Mg) and higher abundance of Bacteroidetes (see Hypo-Mg vs. CTRL and Hyper-Mg) in both untreated and DSS-treated groups (Mann-Whitney U *p*-values < 0.05) (Figure 2, left panels). It was also evident that Mg supplementation in DSS-induced colitis caused a decrease in Proteobacteria and an increase in TM7 (Mann-Whitney U *p*-values < 0.05) (Figure 2, middle and right panels).

**Figure 3:**
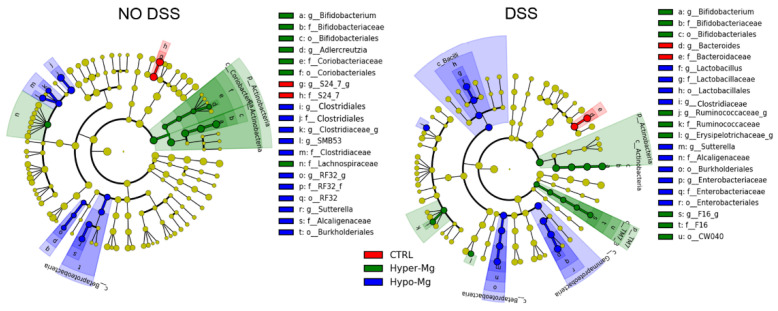
Dietary Mg content modulates gut abundance of specific bacterial taxa. Linear discriminant analysis (LDA) Effect size (LEfSe) identified taxa that differentially characterize Hypo-Mg, Hyper-Mg and CTRL groups in the absence (**left panels**) or presence of colitis (**right panels**).

Linear discriminant analysis (LDA) Effect Size (LEfSe) allows to identify the features most likely to explain differences between groups. In untreated (no DSS) animals, any difference can be ascribed to the effect of dietary Mg. Low Mg availability (Hypo-Mg group) was characterized by high presence of Clostridiales, Clostridiaceae, SMB53, *Sutterella* and RF32. High Mg availability (Hyper-Mg group) resulted in higher abundance of *Bifidobacterium*, *Adlercreutzia* and Lachnospiraceae (Figure 3, left panel and Figure S2A). DSS-induced colitis caused an increase in the following discriminating taxa: Enterobacteriaceae, *Sutterella,* Clostridiaceae and *Lactobacillus* in the Hypo-Mg group; *Bifidobacterium*, F16, and Ruminococcaceae in the Hyper-Mg group; and *Bacteroides* in the CTRL group (Figure 3, right panel and Figure S2B).

#### 3.2.2. Co-Occurrence and Co-Exclusion Patterns

**Figure 4:**
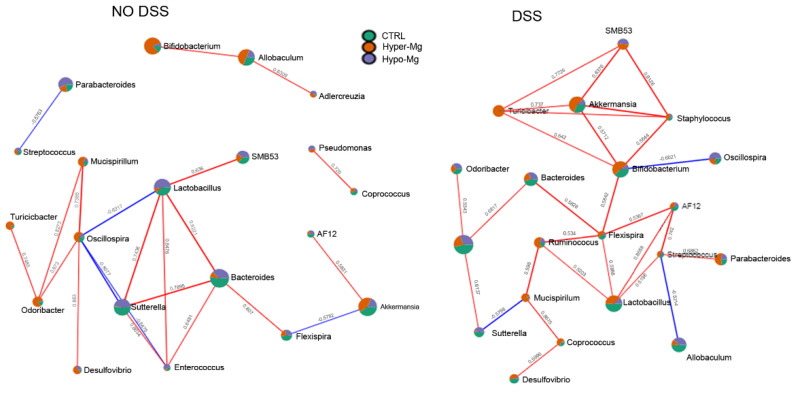
Dietary Mg content modulates gut bacterial interactions. Network analysis of intestinal microbiota using Pearson’s correlation coefficients between diet groups in the absence (**left panels**) or presence of colitis (**right panels**). The nodes represent genera; the edges represent the correlation between genera (blue lines, negative correlations; red lines, positive correlations). Nodes are colored according to their relative abundance in each diet group.

Correlation networks are useful in identifying complex interactions between bacteria. (Figure 4). In our model, in the absence of colitis, the nodes connected by more than three edges included *Oscillospira, Sutterella, Lactobacillus* and *Bacteroides* (Figure 4, left panel).

*Oscillospira*, which was more abundant in the Hyper-Mg group, was negatively correlated (blue lines, co-exclusion patterns) with *Lactobacillus* and *Enterococcus* (which were more abundant in the Hypo-Mg group), as well as with *Sutterella* (present in both CTRL and Hypo-Mg groups). On the other hand, *Oscillospira* was positively correlated (red lines, co-occurrence patterns) with *Mucispirillum* and *Odoribacter* (which were more abundant in the Hyper-Mg group). In addition, *Sutterella* was positively connected with *Lactobacillus, Bacteroides* (more abundant in the CTRL group) and *Enterococcus;* while *Lactobacillus* was positively connected also with SMB53, *Bacteroides* and *Enterococcus.* Lastly, *Bacteroides* was positively correlated with *Flexispira* (more abundant in the CTRL group).

In the presence of colitis, the number of co-exclusion patterns was reduced, and the number of nodes connected by more than three edges was increased, including *Akkermansia*, *Turicibacter*, *Staphylococcus*, *Bifidobacterium*, *Flexispira*, *Lactobacillus* and *Streptococcus* (Figure 4, right panel). *Akkermansia* (which was more abundant in the Hyper-Mg group) showed co-occurrence patterns with SMB53, *Staphylococcus*, *Bifidobacterium* and *Turicibacter,* which were all increased by the Hyper-Mg diet. *Bifidobacterium* was correlated negatively with *Oscillospira* (which was increased by the Hypo-Mg diet) and positively with *Flexispira* (more abundant in the Hyper-Mg group). In turn, *Flexispira* positively connected to *Bacteroides* (more abundant in the CTRL group), *Ruminococcus* (more abundant in the Hyper-Mg group), *Lactobacillus* (more abundant in the CTRL group) and AF12 (more abundant in both Hyper-Mg and CTRL groups).

#### 3.2.3. Correlation between Gut Microbial Taxa and Colitis Severity

**Figure 5:**
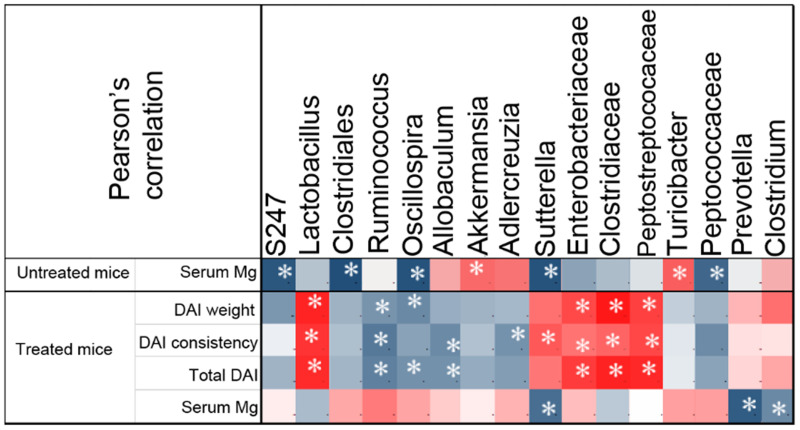
Specific bacterial taxa correlate with disease severity and serum Mg levels. Pearson’s correlation analysis of DAI values, serum Mg levels and abundance of bacterial taxa. Blue and red shades indicate negative or positive correlations, respectively. The asterisk highlights statistically significant correlations (*p*-value < 0.05).

To obtain a measurement of association between microbial taxa and disease activity, we performed Pearson’s correlation analysis of DAI values, serum Mg levels and abundance of specific taxa in all animals (Figure 5). In accordance with our previous work [6], correlation analysis suggested that serum Mg was inversely correlated to disease severity as assessed by either single DAI values or total DAI, although statistical significance was not achieved. In untreated (no DSS) mice, our analysis highlighted a number of significant associations with dietary Mg content. Mg correlated positively with *Akkermansia* and *Turicibacter*; negatively with S247, Clostridiales, *Oscillospira*, *Sutterella* and Peptococcaceae. In the presence of colitis, we found a significant positive correlation between disease severity and *Lactobacillus*, Enterobacteriaceae, Clostridiaceae and Peptostreptococcaceae, and a negative correlation with *Ruminococcus*, *Oscillospira*, *Adlercreuzia* and *Allobaculum*. The only significant associations between serum Mg and represented taxa were negative correlations with *Sutterella*, *Prevotella* and *Clostridium*. However, it is worth noting that most taxa that were significantly associated to disease activity showed an inverse correlation with serum Mg values, although such correlations did not reach statistical significance. As an example, *Lactobacillus* was positively associated with all DAI values, while inversely associated with serum Mg.

#### 3.2.4. Microbial Functional Profiling

**Figure 6:**
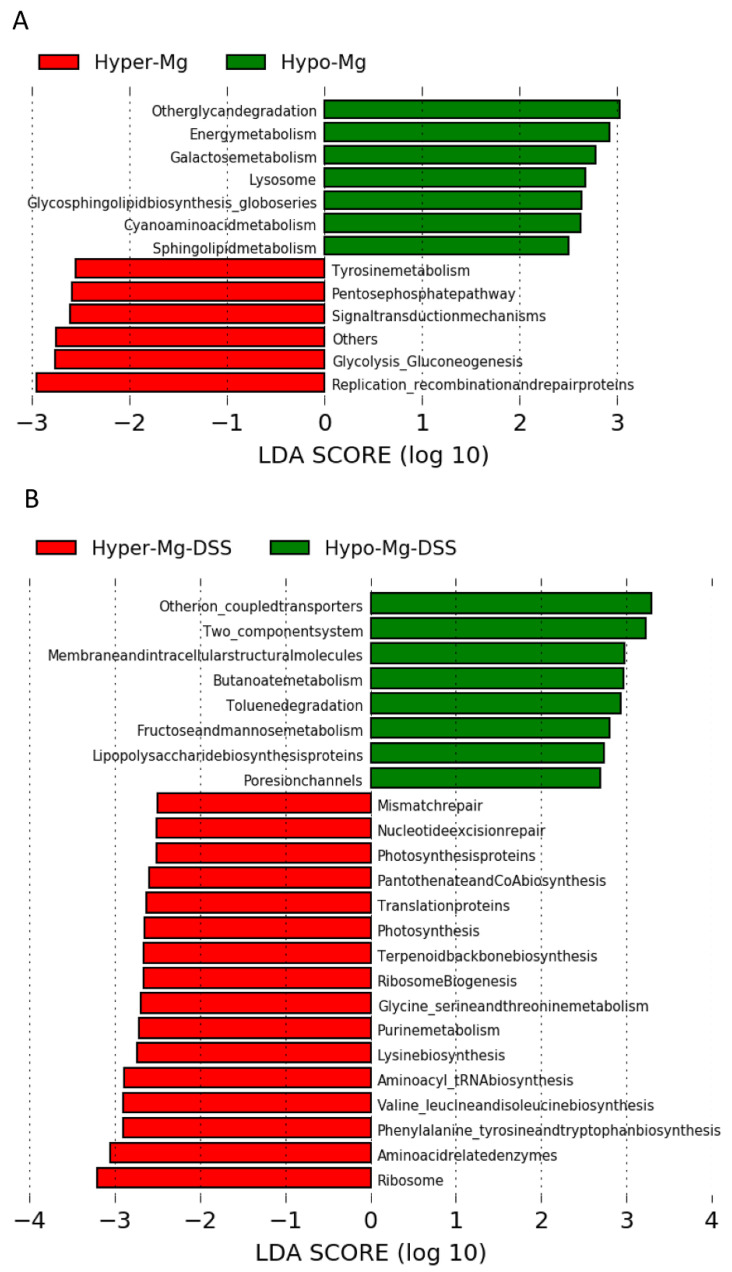
Dietary Mg content modulates specific bacterial functional pathways. PICRUSt-predicted KEGG pathways in the absence (**A**) or presence of colitis (**B**). Linear discriminant analysis (LDA) effect size (LEfSe) was performed on the predicted KEGG pathways. Significance was set to ±2.0, and the log (10)-transformed score is shown to demonstrate effect size.

KEGG pathway predictions were generated by PICRUSt to infer functional differences in gut microbiota related to dietary Mg content in untreated and DSS-treated (colitic) mice (Figure 6). In the absence of colitis, the Mg-deficient microbiome was enriched in KEGG pathways for glycan degradation, oxidative energy metabolism, as well as galactose, glycosphingolipid and sphingolipid metabolism, while the Mg-supplemented microbiome was enriched for pentose phosphate pathway, glycolysis, gluconeogenesis and replication/repair pathways (Figure 6A). In the presence of colitis, in the Hyper-Mg group, KEGG pathways prediction revealed an increase in amino acid and protein synthesis pathways, while in the Hypo-Mg group, LPS synthesis and xenobiotic metabolism pathways were incremented (Figure 6B).

## 4. Discussion

We have previously shown that dietary magnesium supplementation alleviates DSS-induced colitis in mice [6]. Here, we demonstrate that the beneficial effects of Mg are mediated by a modulation of gut microbiota composition, and possibly function. Indeed, α-diversity analysis highlighted the buffering effect of Mg supplementation on gut microbial richness during colitis, in comparison to normal or reduced dietary content of this mineral. Consistently, β-diversity underlined that Mg supplementation produced a distinct microbiota from that of Mg-deficient animals in the presence of colitis.

At the compositional level, independently of colitis, the beneficial Actinobacteria phylum was enriched by Mg supplementation, while Bacteroidetes were more abundant in Mg-deficient conditions.

In the presence of colitis, Mg supplementation increased abundance of TM7 while decreased the presence of the harmful Proteobacteria phylum. Blooming of TM7 could result from the combined effect of high dietary Mg content together with the presence of colitis: in fact, higher abundance of this phylum has been associated with both dietary Mg supplementation in a rat model [15] and human IBD [27].

Amongst Actinobacteria, *Bifidobacterium* abundance was increased in Mg-supplemented mice. Bifidobacteria confer health benefits to the host via their metabolic activities, as they metabolize a wide range of indigestible oligosaccharides to acetic and lactic acids and consequently act as effective scavengers in the large intestine when large quantities of indigestible oligosaccharides are ingested [28]. Bifidobacteria are also known to reduce intestinal LPS and to improve mucosal barrier function [29,30]. *Bifidobacterium* strains were proved to prevent DSS-induced colitis and associated gut microbial dysbiosis in mice [31]. Furthermore, a recent meta-analysis showed that probiotic supplements that are based on *Bifidobacterium* are more likely to be beneficial for IBD remission [32]. During short-term Mg deficiency, a reduction of bifidobacteria in the cecal content of mice has been reported [11], probably because of the important role of Mg in bifidobacteria growth [33,34]. Indeed, the presence of *Bifidobacterium* showed a positive correlation trend with serum Mg and a negative correlation trend with disease activity.

We found a significant positive correlation between systemic Mg availability and *Akkermansia* in the absence of colitis. As mucin is the primary energy source for this microorganism [35], this may reflect an improved mucosal barrier function induced by high Mg conditions. Moreover, it has been reported that administration of *Akkermansia muciniphila* improves DSS-induced colitis in animals models [36,37]. Therefore, we suggest that in our model Mg supplementation could ameliorate colitis symptoms through the increase of this microorganism in the gut. Further experiments are mandatory in order to confirm the active role of dietary magnesium in modulating *A. muciniphila* in health as well as in disease.

In accordance with previous studies [14,38], we found that a low Mg condition is characterized by increased abundance of SMB53 and *Lactobacillus*. In an in vitro study, cultures of *Lactobacillus* spp. increased Mg availability in cheese [39]. Therefore, the increase of *Lactobacillus* in Mg deficiency could represent a compensatory mechanism to increment Mg availability in the intestine [40]. Interestingly, increased abundance of SMB53 and *Lactobacillus* was also found in mice that spontaneously develop obesity and diabetes [41], and several studies have reported that lower magnesium consumption is correlated with an increased risk of insulin resistance [42]. In in vitro IgA-coating experiments, *Lactobacillus* and SBM53 have been recognized as pro-inflammatory pathobionts that could have a role in intestinal diseases such as IBD [43,44].

In IBD, overgrowth of Enterobacteriaceae (which belong to the Proteobacteria phylum) has been associated to dysbiosis in response to inflammation and intestinal permeability [45]. We found that, in the presence of colitis, the abundance of Enterobacteriaceae was a distinctive feature of the Mg-deficient diet, which supports the hypothesis that Mg supplementation might restrain blooming of this pro-inflammatory bacterial family. Mg deficiency may contribute to Enterobacteriaceae overgrowth by two different mechanisms: (1) by activating sensor systems such as PhoP/PhoQ and directly promoting growth [8]; and (2) by creating an oxidative environment [7], which may promote colonization with facultative anaerobic enteric pathogens [46].

Moreover, in the presence of colitis, correlation network analysis evidenced a higher number of correlation nodes involving bacteria favored by the Mg-supplemented diet, which suggests a positive influence of Mg on connection and integration of the gut microbiota ecosystem.

Pearson’s correlation analysis confirmed known associations between colitis severity and bacterial taxa. For example, an increase in *Lactobacillus*, Enterobacteriaceae, Clostridiaceae and Peptostreptococcaceae was associated to worsening of symptoms, while Ruminococcaceae, *Oscillospira*, and *Allobaculum* showed an inverse trend. *Oscillospira* and *Allobaculum* are putative butyrate producers that have a beneficial role in metabolism [47,48]. Particularly, *Oscillospira* resulted significantly reduced in patients with Crohn’s disease [49] and *Allobaculum* was negatively correlated with inflammation in mice [50,51,52]. Interestingly, it was suggested that *Sutterella*, which in our model was negatively correlated with Mg, could contribute to pathogenesis and therapy outcomes in ulcerative colitis [53], probably by its capability to degrade IgA [54].

The beneficial effect of Mg supplementation was also evidenced by KEEG prediction analysis. In the absence of colitis, dietary Mg content seemed to steer energy metabolism towards either oxidative (in low Mg conditions) or glycolytic (in high Mg conditions) pathways. In addition, the Hypo-Mg diet seemed to activate sphingolipid metabolism, and in general, catabolic processes. Sphingolipids have been implicated in neurodegenerative processes, metabolic disorders, cancers, and cardiovascular disorders [55]; moreover, they are potent modulators of the immune response that may enhance or prevent inflammation, depending on the specific molecules and the microenvironment, and are being investigated as therapeutic targets or agents in IBD [56]. In the presence of colitis, Mg supplementation enhanced pathways involved in protein synthesis, DNA repair and translation, while Mg deficit increased xenobiotic metabolism. These findings are strikingly consistent with the well-established biochemical roles of Mg and the close link between Mg availability and biosynthetic metabolism required for cell growth [57,58]. Similarly, low Mg conditions trigger a survival response by activating degradative and oxidative pathways, which may contribute to boost inflammation.

## 5. Conclusions

In conclusion, Mg supplementation affects composition, function, and interplay of gut microbiota in mice. Moreover, in the presence of colitis, this beneficial effect is amplified, leading to the amelioration of clinical symptoms through the increment of bacteria that produce short-chain fatty acids and are involved in intestinal health and metabolic homeostasis. At the same time, high Mg intake reduces bacteria involved in inflammation and human IBD, and consequently reduces harmful metabolism. If confirmed in humans, these findings may represent the basis for developing a safe and cost-effective nutritional intervention for IBD patients aiming at restoring a beneficial intestinal flora, ameliorating symptoms, and reducing cost and side effects of therapies.

## Data Availability

All raw sequences have been archived in NCBI database: PRJNA753047 (https://www.ncbi.nlm.nih.gov/bioproject) (accessed on 1 November 2021).

## Supplementary Materials

The following are available online at https://www.mdpi.com/article/10.3390/nu13124188/s1, Figure S1: Dietary magnesium modulates experimental colitis severity, Figure S2: Dietary Mg content corresponds to different features of gut microbiota.
