# Supplementary material for: Dietary Magnesium Alleviates Experimental Murine Colitis through Modulation of Gut Microbiota
Source: Nutrients. 2021 Nov 23;13(12):4188. doi: 10.3390/nu13124188 (PMC8707433; doi:10.3390/nu13124188)
Supplement: Supplementary file 1 [file nutrients-13-04188-s001.zip › nutrients-1467980-supplementary.pdf]

Supplementary materials

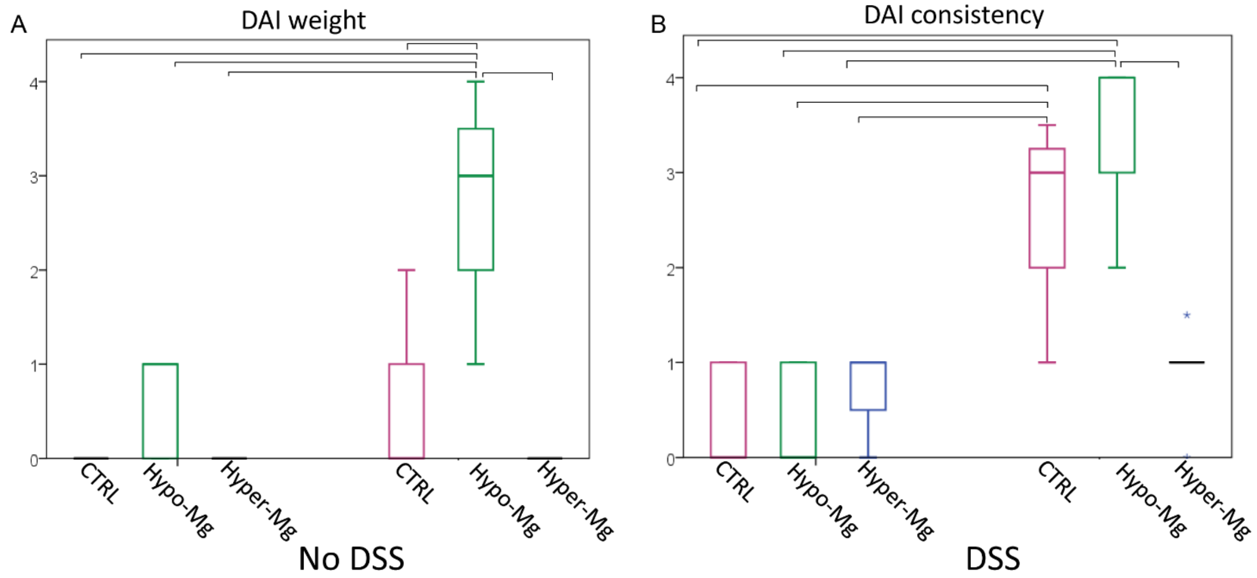

Figure S1. Dietary magnesium modulates experimental colitis severity. Mice were fed an Mg-deficient (Hypo-Mg), normal (CTRL), or Mg-enriched (Hyper-Mg) diet, and colitis was induced by 2.5% DSS administration for 5 days, followed by a recovery period until day 12. Disease activity index (DAI) for weight loss (A) and fecal consistency (B) in untreated and DSS-treated (colitic) mice on the three Mg-adjusted diets at day 12 are shown. Box plots report median, minimum and maximum values, and the 25th and 75th percentile values; any outliers are indicated by stars. Statistical significance (p-value < 0.05) was assessed by one-way ANOVA with Bonferroni-adjusted post-hoc test and indicated by horizontal bars.

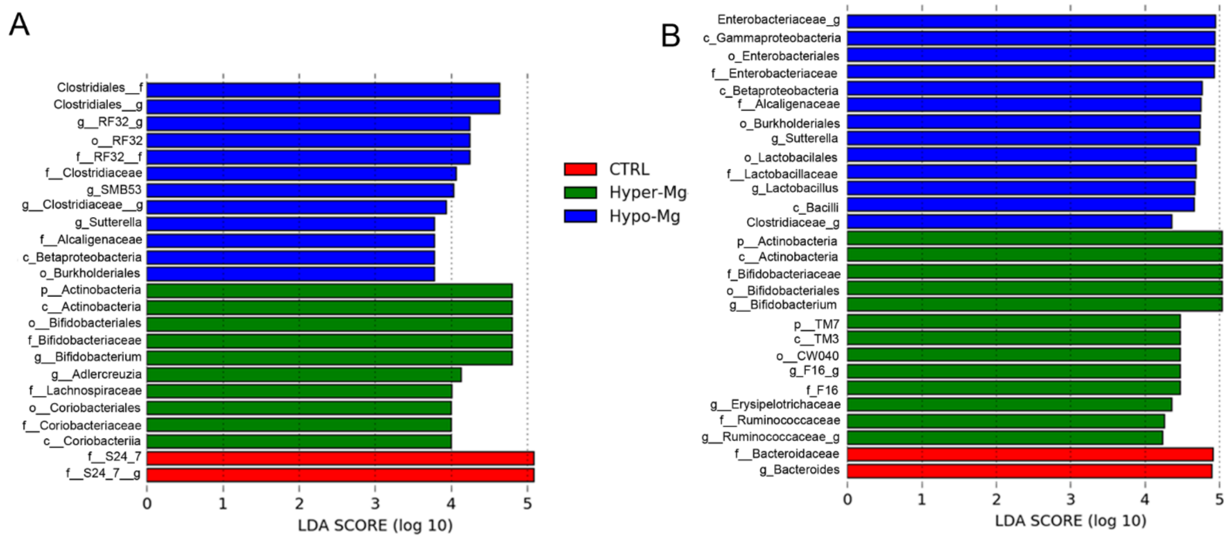

Figure S2. Dietary Mg content corresponds to different features of gut microbiota. The figure reports taxa that differentially characterize Hypo-Mg, Hyper-Mg and CTRL groups in the absence (A) or presence of colitis (B), identified by linear discriminant analysis (LDAS) effect size (LEfSe). An alpha value of 0.05 and an effect size threshold of 2 were used to identify significant taxa.
